# Household food security and HIV status in rural and urban communities in the Free State province, South Africa

**DOI:** 10.1080/17290376.2017.1379428

**Published:** 2017-10-11

**Authors:** Michélle Pienaar, Francois C. van Rooyen, Corinna M. Walsh

**Affiliations:** ^a^ PhD (Nutrition and Dietetics), Department of Nutrition and Dietetics, Faculty of Health Sciences, University of the Free State, Bloemfontein, South Africa; ^b^ MComm, is a researcher and lecturer in the Department of Biostatistics, Faculty of Health Sciences, University of the Free State, Bloemfontein, South Africa; ^c^ PhD (Nutrition and Dietetics), is an associate professor in the Department of Nutrition and Dietetics, Faculty of Health Sciences, University of the Free State, Bloemfontein, South Africa

**Keywords:** HIV, poverty, food security

## Abstract

Higher socioeconomic status impacts profoundly on quality of life. Life-event stressors, such as loss of employment, marital separation/divorce, death of a spouse and food insecurity, have been found to accelerate disease progression among people with human immunodeficiency virus (HIV). The objective of this study was to determine significant independent sociodemographic and food security factors associated with HIV status in people from rural and urban communities in the Assuring Health for All study, which was undertaken in rural Trompsburg, Philippolis and Springfontein and urban Mangaung, in the Free State Province of South Africa. Sociodemographic and food security factors associated with HIV status were determined in 886 households. Logistic regression with forward selection (*p* < 0.05) was used to select significant independent factors associated with HIV status. Variables with a *p*-value of <0.15 were considered for inclusion in the model. Adults 25–64 years of age were eligible to participate. Of the 567 rural participants, 97 (17.1%) were HIV-infected, and 172 (40.6%) of the 424 urban participants. A relatively high percentage of respondents had never attended school, while very few participants in all areas had a tertiary education. The unemployment rate of HIV-infected adults was higher than that of HIV-uninfected adults. A high percentage of respondents in all areas reported running out of money to buy food, with this tendency occurring significantly more among urban HIV-infected than HIV-uninfected respondents. In all areas, a high percentage of HIV-infected respondents relied on a limited number of foods to feed their children, with significantly more HIV-infected urban respondents compared to their uninfected counterparts reporting this. Most participants in all areas had to cut the size of meals, or ate less because there was not enough food in the house or not enough money to buy food. During periods of food shortage, more than 50% of respondents in all areas asked family, relatives or neighbours for assistance with money and/or food, which occurred at a higher percentage of HIV-infected rural participants compared to HIV-uninfected rural participants. More than half of all participants reported feeling sad, blue or depressed for two weeks or more in a row. HIV infection was negatively associated with being married (odds ratio 0.20 in rural areas and 0.54 in urban areas), while church membership decreased the likelihood of HIV (odds ratio 0.22 in rural areas and 0.46 in urban areas). Indicators of higher socioeconomic status (having a microwave oven and access to vegetables from local farmers or shops) decreased the likelihood of HIV in rural areas (odds ratios 0.15 and 0.43, respectively). Indicators of lower socioeconomic status such as spending less money on food in the rural sample (odds ratio 3.29) and experiencing periods of food shortages in the urban sample (odds ratio 2.14), increased the likelihood of being HIV-infected. Interventions aimed at poverty alleviation and strengthening values can contribute to addressing HIV infection in South Africa.

## Introduction

Globally, lower-income populations are most severely affected by human immunodeficiency virus (HIV) infection (Joint United Nations Programme [UNAIDS, 2011]). In addition, gender and age dynamics play a considerable role in the spread of HIV infection (Academy of Science of South Africa [ASSAf], 2007), with strong evidence that socioeconomic inequalities influence the spread of HIV infection (Gillespie, 2008; Piot, 2008).

Poverty is a basic cause of undernutrition, since it is associated with unemployment; inability to pay for food, healthcare and basic services; disintegration of family life; inability to care for children; vulnerability; homelessness and hopelessness (ASSAf, 2007). Therefore, unemployment and poverty are primary concerns for most people living with HIV/AIDS (acquired immune deficiency syndrome) (De Paoli, Mills, & Grønningsæter, 2012). Furthermore, life-event stressors (such as loss of job, marital separation/divorce, death of a spouse and food insecurity), particularly those events that are perceived as severe (Leserman et al., 1999), as well as denial of HIV-infection, have been found to accelerate disease progression among people with HIV (Leserman et al., 2000).

According to the Food and Agriculture Organization (1996), ‘food security exists when all people, at all times, have physical and economic access to sufficient safe and nutritious food that meets their dietary needs and food preferences for an active and healthy life.’ Access to adequate food is threatened for family members in HIV-affected households (Food and Agriculture Organization of the United Nations, 2003). Households experience food insecurity in the most basic sense when their resources are inadequate simply to obtain ‘enough food’ in order to meet basic needs; the condition that in its severe form, results in hunger for household members (Keenan, Olson, Hersey, & Parmer, 2001).

The nature and extent of the impact that the HIV epidemic has on food security are largely unknown (Sibanda, Kalibwani, & Kureya, 2007). It is known, however, that the ability of people affected by the disease to develop and sustain themselves is disrupted (Piot, 2008). The influence of sociodemographic status and household food security on HIV-infection is immense, but remains largely undetermined in the Free State Province of South Africa.

## Objectives

Understanding the various sociodemographic and household food security factors associated with HIV status could provide awareness for and contribute to the development of important interventions. The study investigated the sociodemographic profile and household food security in HIV-infected and HIV-uninfected persons in rural and urban communities in the Free State Province, South Africa. In addition, significant independent sociodemographic and household food security factors associated with HIV status were determined.

## Methods

### Study design, target population and sampling

A cross-sectional study was undertaken. The study formed part of the assuring health for all study that aimed to determine how living in rural and urban communities can influence lifestyle and health. In rural areas all households were eligible to participate. In urban areas, a stratified proportional cluster sample was selected, stratified by area and formal plot/squatter households in open areas. Using randomly selected *X* and *Y* coordinates, 100 starting points were selected in this way. From each point five adjacent starting households were approached to participate in the study. Every adult member of households in both rural and urban communities, who gave informed consent and were between 25 and 64 years of age, were eligible to participate.

### Pilot study

A pilot study was conducted before the main survey and included five persons in each area, similar to the target group, in order to determine whether questions included in the questionnaire could be understood easily, and to determine the amount of time needed to complete the questionnaire. Minor changes (mostly technical editing) were made to the questionnaire after the pilot study.

### Methods and techniques

The venues where data were collected included stations for the collection of blood and urine samples, a food station, a medical examination station, and a station where anthropometric measurements were obtained. Blood specimens were analysed in an accredited laboratory using standard techniques.

The Sociodemographic Questionnaire and Household Food Security and Food Procurement Questionnaire were used to collect data related to sociodemographic profile and food security. These two questionnaires were adapted from those developed for the prospective urban rural epidemiology study (Teo, Chow, Vaz, Rangarajan, & Yusuf, 2009). Hunger was determined by means of the widely used and validated Community Childhood Hunger Identification Project index (Wehler, Scott, & Anderson, 1992). It is based on eight questions that indicate whether adults and children in a household experience food shortages, perceive food insufficiency and alter due to resource limitations or inadequate food resources. Two additional questions related whether the household was experiencing food shortage at the time of the survey and to coping mechanisms employed, were added. A structured interviewing technique was used to complete the questionnaires with one adult member of each household. In very few cases, Sesotho, Setswana and isiXhosa interpreters assisted the researchers.

### Validity and reliability

To assure validity, all questions were related to the objectives of the study and were based on issues discussed in relevant literature. Random samples of 10% of the rural and urban participants were interviewed a second time by the researchers to determine reliability of questions. Where the percentage of given answers to questions differed with more than 20%, the question was considered unreliable. Only one question was found to be unreliable and results are not reported.

### Data collection

Before data were collected, induction meetings for community members and other role players were arranged in each community. The role players included clinic staff, church leaders, community leaders and any members of the community who were interested in learning more about the project or had questions that they wanted to ask.

Data collection took place at different research venues, including the community hall in the rural areas or at the Mangaung University Community Partnership Programme nutrition centre in the urban area.

On days of data collection, identity documents were screened in order to ascertain that participants met the inclusion criteria for age. All stations at each venue needed to be completed in order to be included in the study. Thereafter, questionnaires related to the following were completed: sociodemography and household food security (one of each per household), 24-hour recall, physical activity, and self-reported health status (one of each for every participant).

### Statistical analysis

Descriptive statistics were calculated and included frequencies and percentages (for categorical data) and means and standards deviations (SDs) (for symmetrical numerical variables), or medians and percentiles (for skew numerical variables). Differences between HIV-infected and HIV-uninfected groups were assessed by *p*-values [t-tests (for symmetrical numerical variables), Mann–Whitney tests (for skew numerical variables), chi-squared tests (for categorical variables) or Fisher's extact test (for categorical variables with sparse data)], or 95% confidence intervals (CIs) for median, mean or percentage differences. Logistic regression with forward selection (*p* < 0.05) was used to select significant independent factors associated with HIV status. Variables with a *p*-value of <0.15 were considered for inclusion in the model. Age and gender were entered in each model as possible factors. All analyses were performed by the Department of Biostatistics, UFS.

#### Sociodemographic and household food security factors associated with HIV status in rural participants

The sociodemographic variables considered for inclusion were (i) education (none vs. others); (ii) type of dwelling (brick/concrete, traditional mud, tin corrugated iron, other); (iii) home has a working stove/hot plate (yes vs. no); (iv) home has a working microwave oven (yes vs. no); (v) home has a working television (yes vs. no); (vi) household income per month [R500 (±$39) or less, R501–1000 (±$40–78), R1001–3000 (±$78.4–235), R3001 (±$235.1) and more]; and (vii) marital status (married vs. not). The variables selected in the model were age, marital status and owning a microwave oven. Church membership, experienced violence in last 12 months, experienced death of spouse during past 12 months, experienced unavailability of food/food insecurity during past 12 months and care for orphans in household (all as yes vs. no).

The household food security variables considered for inclusion (i) the amount of money spent on food for household weekly [up to R50 (±$3.9), R51–100 (±$4–7.8), R101 (±$7.9) and more]; (ii) growing vegetables (yes vs. no); (iii) growing green, leafy vegetables (yes vs. no); (iv) growing pumpkins (yes vs. no); (v) growing beans (yes vs. no); (vi) growing beetroot (yes vs. no); (vii) owning livestock (yes vs. no); (viii) fruits easily available from local farmers/shops (yes vs. no); (ix) vegetables easily available from local farmers and shops (yes vs. no); (x) ever eating less than you should because there was not enough money for food (yes vs. no); (xi) children ever eating less than you felt they should because there was not enough money for food (yes vs. no); (xi) any of the children ever saying they were hungry because there was not enough food in the house (yes vs. no); (xii) ever cutting the size of children's meals or children skipping meals because there was not enough money to buy food (yes vs. no). The variables that were selected in the model included age, money spent on food for the household weekly, and vegetables being easily available from local farmers and shops.

Thereafter the variables which were significant in the socioeconomic and household food security models – age, marital status, possession of a microwave oven, money weekly spent on food for the household, whether vegetables were easily available from local farmers and shops – were considered for the final model.

#### Sociodemographic and household food security factors associated with HIV status in urban participants

The sociodemographic variables considered for inclusion were (i) education (none vs. others); (ii) employment status (unemployed vs. not); (iii) home has working refrigerator/freezer (yes vs. no); (iv) household income [R500 ($39) or less, R501–1000 ($40–78), R1001–3000 ($78.4–235), R3001 ($235.1) and more]; and (v) marital status (married vs. other). The variables selected in the model were age and marital status. Church membership, experienced violence in last 12 months, experienced death of spouse during past 12 months, experienced unavailability of food/food insecurity during past 12 months and care for orphans in household (all as yes vs. no).

The household food security variables considered for inclusion were (i) money spent on food for household weekly [up to R50 (±$3.9), R51–100 (±$4–7.8), R101 (±$7.9) and more]; (ii) growing cabbage (yes vs. no); (iii) growing green/leafy vegetables (yes vs. no); (iv) growing beans (yes vs. no); (iv) the household ever running out of money to buy food (yes vs. no); (v) relying on a limited number of foods to feed the children (yes vs. no); ever eating less than enough because of a shortage of money for food (yes vs. no); children ever eating less than you felt they should because of insufficient money for food (yes vs. no); (vi) children ever going to bed hungry because of insufficient money to buy food (yes vs. no); (vii) children ever saying they were hungry because of insufficient food in the house (yes vs. no); and (viii) the family ever experiencing periods of food shortage (yes vs. no). The following selected in the model included age, ever experiencing periods of food shortage, and growing green/leafy vegetables.

Thereafter the variables that were significant in the socioeconomic and household food security models (namely age, marital status, whether the family ever experiencing periods of food shortage, and growing green/leafy vegetables) were considered for the final model. Age, marital status and the family ever experiencing periods of food shortage were selected in the model with odds ratios.

### Ethical considerations

The study was approved by the Ethics Committee of the Faculty of Health Sciences at the UFS (ETOVS 21/07), as well as the Free State Department of Health and local municipalities. The researchers obtained written informed consent from all participants in their language of choice.

## Results

### Sociodemographic information

The study group comprised 570 rural and 426 urban participants. Of the 570 rural participants, 567 had HIV results and 97 (17.1%) were HIV-infected. Of the 426 urban participants, 424 had HIV results and 172 (40.6%) were HIV-infected. Forty-three of these 172 participants (25.0%) were on antiretroviral therapy, compared to only four (4.1%) in rural areas.

HIV-infected rural participants were significantly younger (median age 40.5 years; range 27-65) than HIV-uninfected rural participants (median age 51 years; range 25-65) (*p* = 0.001). Similar results were found in the urban sample, with HIV-infected respondents having a median age of 38 years (range 25-63) and HIV-uninfected respondents a median age of 49 years (range 25-64) (*p* = 0.0001). In all areas, a larger percentage of participants tended to be female and significantly more HIV-uninfected people were married compared to HIV-infected participants (Table 1). A relatively high percentage of respondents had never attended school (17.8% HIV-infected and 28.0% HIV-uninfected participants in the rural sample; 11.8% HIV-infected and 22.1% HIV-uninfected participants in the urban sample); while very few participants in all areas had a tertiary education. In the urban area, the unemployment rate of HIV-infected adults was significantly higher than in HIV-uninfected adults (62.8% vs. 49.4%, *p* = 0.007). The percentage of full-time wage earners was low in all groups.

**Table 1.** Sociodemographic information.

Variable	Rural					Urban				
	HIV+		HIV−		*p*-Value^a^	HIV+		HIV−		*p*-Value^b^
	*n*	%	*N*	%		*n*	%	*n*	%	
Gender										
RHP 91, RHN 451 UHP 168, UHN 247										
Male	25	32.6	88	19.5	0.09	37	22.0	62	25.1	0.47
Female	66	72.5	363	80.5		131	78.0	185	74.9	
Marital status										
RHP 89, RHN 451 UHP 162, UHN 244										
Child	1	1.12	2	0.4		0	0.0	0	0.0	
Never married	32	36.0	80	17.7		70	43.2	69	28.3	
Married/traditional marriage	9	10.1	168	37.3	0.0001*	31	19.1	89	36.5	0.0002*
Living with partner	17	19.1	54	12.0		23	14.2	18	7.4	
Widowed	15	16.9	86	19.1		23	14.2	35	14.3	
Separated	11	12.4	43	9.5		6	3.7	12	4.9	
Divorced	4	4.5	17	3.8		8	4.9	21	8.6	
Other	0	0	1	0.2		1	0.6	0	0	
Level of education										
RHP 90, RHN 439; UHP 169, UHN244										
None	16	17.8	123	28.0		20	11.8	54	22.1	
Primary school	34	37.8	134	30.5		62	36.7	87	35.7	
Gr. 8–10	21	23.3	117	26.7	0.12	43	25.4	56	23.0	0.14
Gr. 11–12	18	20.0	59	13.4		37	21.9	44	18.0	
Tertiary education	1	1.1	2	0.5		1	0.6	2	0.8	
Employment status										
RHP 93, RHN 466; UHP 172, UHN 251										
Housewife by choice	1	1.1	14	3.0		1	0.6	2	0.8	
Unemployed	27	29.0	107	22.9	0.21	108	62.8	124	49.4	0.007*
Self-employed	2	2.2	7	1.5		1	0.6	3	1.2	
Full-time wage earner (receives a salary)	5	5.4	37	8.0		7	4.1	14	5.6	
Part-time/piece job	58	62.4	301	64.6		55	32.0	108	43.0	
Not applicable, for example, deceased	0	0	0	0		0	0	0	0	

Notes: RHP = rural, HIV-positive; RHN = rural, HIV-negative; UHP = urban, HIV-positive; UHN = urban, HIV-negative.

^a^ *p*-Value for difference between HIV-positive and HIV-negative rural participants using chi-squared or Fisher's exact test, as appropriate.

^b^ *p*-Value for difference between HIV-positive and HIV-negative urban participants using chi-squared or Fisher's exact test, as appropriate.

*Statistically significant difference.

Table 2 summarises the housing characteristics of participants. In all areas, most households reported living in brick houses. A higher percentage of HIV-infected urban participants (21.5%) lived in an overcrowded house (≥ 2.5 median room density) compared to HIV-uninfected urban participants (16.4%). Few urban participants reporting having a bathroom in the house (8.7% HIV-infected and 17.1% HIV-uninfected participants, *p* = 0.01). A statistically significantly lower percentage of HIV-infected rural participants owned a refrigerator and/or freezer (47.3%) than HIV-uninfected rural respondents (64.4%) (*p* = 0.002). A similar trend was found with regard to owning a microwave oven (6.5% and 23.6%, respectively; *p* = 0.002) and television (38.7% and 59.0%, respectively; *p* = 0.0005). In both rural and urban areas, the majority of households had their own tap and almost all households used it as the main source of drinking water. Most households reported having a flush type toilet.

**Table 2.** Housing features.

Variable	Rural (*n* = 539)					Urban (*n* = 423)				
	HIV+ (*n* = 93)		HIV − (*n* = 466)		*p*-Value^a^	HIV+ (*n* = 172)		HIV− (*n* = 251)		*p*-Value^b^
	*n*	%	*n*	%		*n*	%	*n*	%	
Type of dwelling										
Brick, concrete	73	78.5	394	84.6	0.15	142	82.6	210	83.7	0.76
Traditional mud	0	0	2	0.4		0	0	0	0	
Corrugated iron	20	21.5	67	14.4		30	17.4	40	15.9	
Plank, wood	0	0	0	0		0	0	0	0	
Other	0	0	3	0.6		0	0	1	0.4	
Median room density										
<2.5 (not overcrowded)	82	88.2	414	88.8	0.85	135	78.5	210	83.7	0.17
≥2.5 (overcrowded)	11	11.8	52	11.8		37	21.5	41	16.4	
* *Bathroom in house	19	20.4	91	19.5	0.84	15	8.7	43	17.1	0.01*
Bathroom outside	2	2.2	19	4.1	0.55	152	88.4	209	83.3	0.01
Kitchen or cooking area in house	88	94.6	446	96.0	0.58	169	98.3	246	98.0	1.0
Has electricity	83	89.3	438	94.2	0.079	147	85.5	222	88.5	0.36
Home has a working										
Refrigerator and/or freezer	44	47.3	300	64.4	0.002*	116	67.4	188	74.9	0.09
Stove (gas, coal or electric)	66	70.9	368	79.0	0.08	137	79.7	209	83.3	0.34
Primus or paraffin stove	54	58.0	252	54.1	0.48	83	48.3	119	47.4	0.86
Microwave oven	6	6.5	110	23.6	0.002*	66	38.4	98	39.0	0.88
Radio	68	73.1	361	77.5	0.36	139	80.8	208	82.9	0.58
Television set	36	38.7	275	59.0	0.0005*	116	67.4	176	70.1	0.55
Main source of drinking water										
Own tap	88	94.6	449	96.4	0.39	132	76.7	201	80.1	0.41
Communal tap	4	4.3	15	3.2		39	22.7	47	18.7	
River, dam	0	0	1	0.2		0	0	0	0	
Borehole, well	0	0	0	0		0	0	2	0.8	
Other	1	1.1	1	0.2		1	0.6	1	0.4	
Type of toilet in household										
Flush	86	92.5	440	94.6	0.42	143	83.1	221	88.1	0.15
Pit	0	0	3	0.7		5	2.9	7	2.8	
Bucket, pot	2	2.2	0	0		20	11.6	21	8.4	
VIP	0	0	15	3.2		2	1.2	2	0.8	
Other (neighbour's toilet)	5	5.4	7	1.5		2	1.2	0	0	

^a^ *p*-Value for difference between HIV-positive and HIV-negative rural participants using chi-squared or Fisher's exact test, as appropriate.

^b^ *p*-Value for difference between HIV-positive and HIV-negative urban participants using chi-squared or Fisher's exact test, as appropriate.

*Statistically significant difference.

In the majority of households one or two people contributed to the household income (Table 3). In rural areas, household income differed significantly between HIV-infected respondents and HIV-uninfected respondents (*p* = 0.005). More HIV-infected rural participants (24.7%) received an income between R100 and R500 (±$7.8–39) compared to their uninfected counterparts (10.7%). The same trend was seen in the urban group, although the difference in income among urban HIV-infected and HIV-uninfected participants was not significant (Table 3). In both areas the percentage of respondents with wages and salaries from formal employment was relatively low. A high percentage of both groups received an old age pension or state grant. As shown in Table 3, the expenditure on food in rural areas differed significantly between HIV-infected and HIV-uninfected participants (*p* = 0.004). A larger percentage of HIV-infected rural respondents (22.2%) spent R50 (±$3.9) or less on food for the household per week, compared to HIV-uninfected participants (8.8%). Very few rural respondents spent more than R201 (±$15.7) per week on food for the household. A similar statistically significant pattern was reported in urban areas (*p* = 0.04).

**Table 3.** Income and expenditure on food.

Variable	Rural					Urban				
	HIV+		HIV−		*p*-Value^a^	HIV+		HIV−		*p*-Value^b^
	*n*	%	*N*	%		*n*	%	*n*	%	
Number of people that contribute to income										
RHP 93, RHN 465; UHP 172, UHN 251										
0	2	2.2	11	2.4	0.92	9	5.2	12	4.8	0.76
1	40	43.0	183	39.3		58	33.7	94	37.5	
2	32	34.4	190	40.8		58	33.7	62	24.7	
3	17	18.2	59	12.7		22	12.8	49	19.5	
4	0	0	14	3.0		16	9.3	19	7.6	
5	0	0	5	1.1		6	3.5	10	4.0	
6	2	2.2	3	0.7		2	1.2	4	1.6	
7	0	0	1	0.2		0	0	0	0	
8	0	0	0	0		1	0.6	1	0.4	
Household income per month^c^										
RHP 93, RHN 466; UHP 172, UHN 251										
None	1	1.1	7	1.5	0.005*	5	2.9	6	2.4	0.081
R100–500 ($7.8–39)	23	24.7	50	10.7		50	29.1	48	19.1	
R501–1000 ($40–78)	32	34.4	168	36.0		46	26.7	75	29.9	
R1001–3000 ($78.4–235)	31	33.3	212	45.5		60	34.9	104	41.4	
R3001–5000 ($235.1–391.5)	2	2.2	17	3.7		3	1.7	7	2.8	
Over R5000 ($391.5)	3	3.2	7	1.5		2	1.2	5	2.0	
Do not know	1	1.1	5	1.1		6	3.5	6	2.4	
Money spent on food for the household weekly^c^										
RHP 90, RHN 457; UHP 166, UHN 216										
R0–50 ($0–3.9)	20	22.2	40	8.8	0.004*	23	13.9	22	8.9	0.04*
R51–100 ($4–7.8)	30	33.3	143	31.3		27	16.3	30	12.1	
R101–150 ($7.9–11.7)	13	14.4	139	30.4		18	10.8	37	15.0	
R151–200 ($11.8–15.7)	12	13.3	66	14.4		12	7.2	21	8.5	
R201–250 ($15.8–19.6)	6	6.7	27	5.9		19	11.5	29	11.7	
R251–300 ($19.7–23.5)	0	0	11	2.4		7	4.2	11	4.5	
R301–350 ($23.6–27.4)	2	2.2	4	0.9		3	1.8	5	2.0	
R351–400 ($27.5–31.3)	2	2.2	8	1.7		0	0	5	2.0	
Over R400 ($31.3)	2	2.2	6	1.3		11	6.3	24	9.7	
Do not know	2	2.2	13	2.8		46	27.7	63	25.5	
Main source of income of household										
RHP 90, RHN 457; UHP 165, UHN 247										
Wages/salaries from formal employment	8	8.9	45	9.9		12	7.3	21	8.5	
Self-employment, including home enterprises	0	0	7	1.5		0	0	0	0	
Casual employment (agricultural or non-agricultural)	13	14.4	50	10.9		22	13.3	15	6.1	
Crop production, livestock sales	0	0	0	0		0	0	3	1.4	
Sale of assets	0	0	0	0		0	0	0	0	
Land/flats/equipment rental	0	0	0	0		0	0	0	0	
Old-age pension or state grant	59	65.6	308	67.4	0.73	85	51.5	117	47.4	0.41
Domestic work	1	1.1	1	0.2		0	0	2	0.8	
Other	9	10.0	46	10.1		46	27.9	89	36.0	

^a^ *p*-Value for difference between HIV-positive and HIV-negative rural participants using chi-squared or Fisher's exact test, as appropriate.

^b^ *p*-Value for difference between HIV-positive and HIV-negative urban participants using chi-squared or Fisher's exact test, as appropriate.

^c^South African Rand vs. US Dollar: R12.77 = $1.

*Statistically significant difference.

Less than half of all participants grew vegetables. Table 4 shows that significantly more HIV-uninfected rural participants grew beans than HIV-infected rural participants, while significantly more HIV-infected urban respondents grew cabbage than HIV-uninfected respondents. Potatoes were the crop most often grown. In terms of owning livestock, a higher percentage of HIV-uninfected respondents reported owning livestock compared to their HIV-infected counterparts, but these differences were not statistically significant. Most respondents reported fairly high availability of fruit and vegetables from local farmers and shops. Significantly more HIV-uninfected rural participants reported that vegetables were easily available than HIV-infected participants (Table 4).

**Table 4.** Food production, preservation and the availability of vegetables, crops and fruit trees.

Variable	Rural					Urban				
	HIV+		HIV−		*p*-Value^a^	HIV+		HIV−		*p*-Value^b^
	*n*	%	*n*	%		*n*	%	*n*	%	
Vegetables grown										
RHP 90, RHN 457; UHP 167, UHN 247										
Yes	31	34.4	207	45.3	0.06	67	40.1	86	34.8	0.27
No	59	66.2	250	54.7		100	59.9	161	65.2	
Type of vegetables produced										
RHP 31, RHN 207; UHP 67, UHN 86										
Cabbage	11	35.5	66	31.9	0.57	18	26.9	12	14.0	0.02*
Carrots	22	71.0	127	61.4	0.51	12	17.9	22	25.6	0.53
Green, leafy vegetables	25	80.7	176	85.0	0.05	66	98.5	80	93.0	0.13
Pumpkin	12	38.7	87	42.0	0.19	15	22.4	18	20.3	0.53
Beans	8	25.8	84	40.6	0.02*	15	22.4	16	18.6	0.10
Beetroot	18	58.1	127	61.4	0.12	20	29.9	26	30.2	0.64
Crops grown										
RHP 90, RHN 457; UHP 167, UHN 247										
Yes	21	23.3	107	23.4	0.98	14	8.4	19	7.7	0.79
No	69	76.7	350	76.6		153	91.6	228	92.3	
Fruit trees owned										
RHP 90, RHN 457; UHP 167, UHN 247										
Yes	61	67.8	319	69.8	0.76	68	40.7	107	43.3	0.59
No	29	32.2	138	30.2		99	59.3	140	56.7	
Livestock owned										
RHP 90, RHN 456; UHP 167, UHN 247										
Yes	14	15.6	113	24.8	0.05	8	4.8	18	7.3	0.31
No	76	84.4	343	75.2		159	95.2	229	92.7	

Notes: RHP = rural, HIV-positive; RHN = rural, HIV-negative; UHP = urban, HIV-positive; UHN = urban, HIV-negative.

^a^ *p*-value for difference between HIV-positive and HIV-negative rural participants using chi-squared or Fisher's exact test, as appropriate.

^b^ *p*-value for difference between HIV-positive and HIV-negative urban participants using chi-squared or Fisher's exact test, as appropriate.

*Statistically significant difference.

As shown in Table 5, a high percentage of respondents in all areas reported running out of money to buy food, with this tendency occurring significantly more among urban HIV-infected than HIV-uninfected respondents. In all areas, a high percentage of HIV-infected respondents relied on a limited number of foods to feed their children, with significantly more HIV-infected urban respondents compared to their uninfected counterparts. Most participants in all areas had to cut the size of meals, or ate less because there was not enough food in the house or not enough money to buy food.

**Table 5.** Prevalence of hunger and coping strategies.

Variable	Rural					Urban				
	HIV+ (*n* = 90)		HIV− (*n* = 456)		*p*-Value^a^	HIV+ (*n* = 166)		HIV− (*n* = 246)		*p*-Value^b^
	*n*	%	*n*	%		*n*	%	*n*	%	
Fruit easily available										
Yes	75	83.3	409	89.7	0.08	147	88.6	225	91.5	0.34
No	15	16.7	47	10.3		19	11.4	21	8.5	
Vegetables easily available										
Yes	72	80.0	412	90.4	0.004*	147	88.6	226	91.	0.25
No	18	20.3	44	9.6		19	11.4	20	8.1	

^a^ *p*-Value for difference between HIV-positive and HIV-negative rural participants using chi-squared or Fisher's exact test, as appropriate.

^b^ *p*-Value for difference between HIV-positive and HIV-negative urban participants using chi-squared or Fisher's exact test, as appropriate.

*Statistically significant difference.

In all areas, a larger percentage of households with HIV-infected participants reported that children had to eat less because there was not enough money, and the difference was significant in rural areas (*p* = 0.009). Significantly more HIV-infected respondents than HIV-uninfected respondents reported that children said that they were hungry because of insufficient food in the house. More HIV-infected rural participants reported cutting the size of the children's meals or skipping meals than HIV-uninfected rural participants. Although the difference did not reach statistical significance, it did indicate a trend (or it was close to significant). Significantly more HIV-uninfected urban respondents reported that their children did not go to bed hungry compared to HIV-infected urban respondents (*p* = 0.04).

Although a large percentage of rural respondents reported periods of food shortage, but in the urban area it was much higher with more HIV-infected respondents reporting periods of food shortage compared to HIV-uninfected respondents. During periods of food shortage, more than 50% of respondents in all areas asked family, relatives or neighbours for assistance with money and/or food, with a higher percentage for HIV-infected rural participants than HIV-uninfected rural participants. HIV-infected rural respondents were more likely to work for payment in kind (15.8%) compared to HIV-uninfected rural respondents (5.7%).

A high percentage of all respondents in this sample attended church (Table 6). A significantly higher percentage of HIV-uninfected respondents were members of a church compared to HIV-infected respondents: 98.2% vs. 93.3% in rural areas (*p* = 0.01) and 92.1% vs. 84.4% in urban areas (*p* = 0.01). Although experience of permanent stress was reported by a higher percentage of HIV-infected participants than HIV-uninfected participants in both groups: 9.1% vs. 8.8% in rural areas and 33.5% vs. 28.7% in urban areas, the difference was not significant. Significantly more HIV-infected urban participants reported loss of a job (43.3%) compared to HIV-uninfected participants (29.9%) (*p* = 0.005). The incidence of violence (27.6% vs. 18.1%, *p* = 0.04) and death of a spouse (12.6% vs. 4.9%, *p* = 0.006) was significantly higher in HIV-infected rural respondents compared to HIV-uninfected rural respondents. Overall, HIV-infected respondents were more likely to have experienced business failure, household break-in, marital separation or divorce, intra-family conflict, major personal injury or illness and violence. In both groups food insecurity was reported in a higher percentage of HIV-infected participants compared to HIV-uninfected participants: 46% vs. 35% in rural areas (*p* = 0.05) and 64% vs. 59.4% in urban areas (*p* = 0.34).

**Table 6.** Church membership and stress.

Variable	Rural					Urban				
	HIV+		HIV−		*p*-Value^a^	HIV+		HIV−		*p*-Value^b^
	*n*	%	*n*	%		*n*	%	*n*	%	
Household run out of money to buy food										
RHP 90, RHN 457; UHP 167, UHN 247										
Yes	61	67.8	338	74.0	0.36	154	92.2	210	85.0	0.03*
No	29	32.2	119	26.0		13	7.8	37	15.0	
Rely on a limited number of foods to feed children										
RHP 63, RHN 342; UHP 134, UHN 210										
Yes	28	44.4	125	36.6	0.23	113	84.3	158	75.2	0.04*
No	35	55.6	217	63.5		21	15.7	52	24.8	
Cut the size of meals or skip any because there is not enough food in house										
RHP 89, RHN 457; UHP 167, UHN 247										
Yes	63	70.8	325	71.1	0.95	132	79.0	183	74.1	0.24
No	26	29.2	132	28.9		35	21.0	64	25.9	
Eat less than you should because there is not enough money for food										
RHP 90, RHN 457; UHP 167, UHN 247										
Yes	58	64.4	261	57.1	0.19	134	80.2	183	74.1	0.14
No	32	35.6	196	42.9		33	19.8	64	25.9	
Participant or children eat less than they feel they should because there is not enough money for food										
RHP 62, RHN 342; UHP 132, UHN 206										
Yes	24	38.7	91	26.6	0.05	103	78.0	150	72.8	0.28
No	38	61.3	251	73.4		29	22.0	56	27.2	
Children say they are hungry because there is not enough food in the house										
RHP 62, RHN 342; UHP 133, UHN 206										
Yes	22	35.5	70	20.5	0.009*	103	77.4	139	67.5	0.04*
No	40	64.5	272	79.5		30	22.6	67	32.5	
Cut the size of your children's meals or skip meals because there is not enough money to buy food										
RHP 62, RHN 342; UHP 132, UHN 206										
Yes	18	29.0	61	17.8	0.05	101	76.5	147	71.4	0.29
No	44	71.0	281	82.2		31	23.5	59	28.6	
Children ever go to bed hungry because there is not enough money to buy food										
RHP 90, RHN 457; UHP 167, UHN 247										
Yes	10	16.1	35	10.3	0.18	92	68.2	119	57.2	0.04*
No	52	83.9	306	89.7		43	31.9	89	42.8	
No children in household	28	31.1	115	25.2		32	19.1	39	15.8	
Family ever experienced periods of food shortage										
RHP 90, RHN 457; UHP 167, UHN 247										
Yes	38	42.2	217	47.5	0.36	145	86.8	188	76.1	0.07
No	52	57.8	240	52.5		22	13.2	59	23.9	
Coping skills during periods of food shortage										
RHP 38, RHN 212; UHP 145, UHN 188										
Found other/additional sources of income	1	2.3	9	4.3		7	4.8	9	4.8	1
Asked family/relatives/neighbours for help (money/food)	24	63.2	115	54.3		86	59.3	112	59.6	24
Family members went to live elsewhere	0	0	1	0.5		23	15.9	32	17.0	0
Sold assets	0	0	0	0		0	0	0	0	0
Worked for payment in kind	6	15.8	12	5.7		1	0.7	0	0	6
Depended on charity/welfare	0	0	1	0.5		2	1.4	2	1.1	0
Borrowed money/ food	7	18.4	48	22.6		17	11.7	30	16.0	7
Increased production of food	0	0	3	1.4		0	0	0	0	0
Could not do anything	0	0	6	2.8		6	4.1	3	1.6	0
Other (credit at store/family members bring food)	0	0	17	8.0		3	2.1	0	0	0
Family member(s) served first										
RHP 90, RHN 457; UHP 167, UHN 247										
Father/men in the family.	12	13.3	65	14.2		33	19.8	53	21.5	
Mother/women in the family	13	14.4	44	9.3		9	5.4	10	4.1	
Children	5	5.6	34	7.4		34	20.4	53	21.5	
All eat at the same time	46	51.1	282	61.7		78	46.7	112	45.3	
Lives and eats alone	14	15.6	32	7.0		13	7.8	19	7.7	

Notes: RHP = rural, HIV-positive; RHN = rural, HIV-negative; UHP = urban, HIV-positive; UHN = urban, HIV-negative.

^a^ *p*-Value for difference between HIV-positive and HIV-negative rural participants using chi-squared or Fisher's exact test, as appropriate.

^b^ *p*-Value for difference between HIV-positive and HIV-negative urban participants using chi-squared or Fisher’s exact test, as appropriate.

*Statistically significant difference.

More than half of all participants reported feeling sad, blue or depressed for two weeks or more in a row. In rural areas 19.3% of HIV-infected and 28.7% of HIV-uninfected participants cared for orphans in their household. In the urban sample 21.7% of HIV-infected and 30% HIV-uninfected participants cared for orphans.

### Sociodemographic and household food security factors associated with HIV status

In addition to descriptive comparisons between HIV-infected and HIV-uninfected participants, logistic regression was used to identify significant sociodemographic and household food security factors associated with HIV status.

#### Sociodemographic and household food security factors associated with HIV status in rural participants

In the rural sample, for every year that age increased, the odds of having HIV decreased by 8%. In this rural sample, HIV infection was negatively associated with having a microwave oven, having access to vegetables from local farmers or shops, and being married. On the other hand, HIV infection was positively associated with spending less than R50 ($3.9) on food per week (odds ratio 3.29) or spending less than R100 ($7.8) on food per week (odds ratio 1.22). Church membership was negatively associated with HIV-infection (odds ratio 0.22), and positively associated with having experienced death of a spouse during the past year (odds ratio 4.91) (Tables 7 and 8).

**Table 7.** Sociodemographic and household food security factors associated with HIV status of rural participants.

Variable	Rural					Urban				
	HIV+		HIV−			HIV+		HIV−		
	*n*	%	*n*	%	*p*-Value^a^	*N*	%	*n*	%	*p*-Value^b^
Church membership										
Yes RHP 89, RHN 452; UHP 160, UHN 240	83	93.3	444	98.2	0.01*	135	84.4	221	92.1	0.01*
Experienced stress										
RHP 88, RHN 443, UHP 164, UHN 244										
Never	30	34.1	148	33.4		25	15.2	31	12.7	
A few periods of stress	30	34.1	139	31.4		51	31.1	80	32.8	
Several periods of stress	20	22.7	117	26.4		33	20.1	63	25.8	
Permanent stress	8	9.1	39	8.8	0.93	55	33.5	70	28.7	0.29
Experienced during past 12 months										
Loss of job RHP 88, RHN 449, UHP 164, UHN 244	14	15.9	60	13.4	0.52	71	43.3	73	29.9	0.005*
Retirement RHP 88, RHN 449, UHP 164, UHN 244	9	10.2	46	10.2	0.99	13	7.9	16	6.6	0.59
Business failure RHP 88, RHN 450, UHP 164, UHN 244	18	20.5	79	17.6	0.51	10	6.1	16	6.6	0.85
Household break in RHP 88, RHN 449, UHP 164, UHN 244	9	10.2	34	7.6	0.40	36	22.0	52	21.3	0.87
Marital separation/ divorce RHP 87, RHN 450, UHP 164, UHN 244	6	6.9	22	4.9	0.43	10	6.1	13	5.3	0.74
Intra-family conflict RHP 88, RHN 447, UHP 162, UHN 242	19	21.6	96	21.5	0.98	43	26.5	50	20.7	0.16
Major personal injury or illness RHP 87, RHN 448, UHP 164, UHN 244	33	37.9	135	30.1	0.15	55	33.5	78	32.0	0.74
Violence RHP 87, RHN 448, UHP 164, URN 244	24	27.6	81	18.1	0.04*	40	24.4	41	16.8	0.059
Death of a spouse RHP 87, RHN 449, UHP 164, UHN 244	11	12.6	22	4.9	0.006*	13	7.9	12	4.9	0.21
Death or major illness of another family member RHP 85, RHN 449, UHP 162, UHN 244	42	49.4	224	49.9	0.93	109	66.5	149	61.1	0.26
Wedding of family member RHP 87; RHN 450, UHP 164, UHN 244	18	20.7	77	17.1	0.42	51	31.1	80	32.8	0.72
New job RHP 87; RHN 448, UHP 164, UHN 244	6	6.9	55	12.3	0.14	17	10.4	23	9.4	0.75
Birth in the family RHP 87, RHN 449, UHP 164, UHN 244	25	28.7	133	29.6	0.86	57	34.8	96	39.3	0.34
Separation from family RHP 87, RHN 449, UHP 164, UHN 244	4	4.6	28	6.2	0.55	43	26.2	50	20.5	0.17
Food insecurity RHP 87, RHN 448, UHP 164, UHN 244	40	46.0	157	35.0	0.05	105	64.0	145	59.4	0.34
Other major stress RHP 89, RHN 452, UHP 154, UHN 230	12	13.5	66	14.6	0.78	30	19.5	49	21.3	0.66
Felt sad, blue or depressed for two weeks or more in a row during past 12 months										
RHP 87, RHN 445, UHP 162, RHN 243	46	52.9	211	47.4	0.35	110	67.9	162	66.7	0.79
Care for orphans in household										
RHP 88, RHN 449, UHP 161, UHN 240	17	19.3	129	28.7	0.06	35	21.7	72	30.0	0.07

^a^ *p*-Value for difference between HIV+ and HIV− rural participants using Chi-squared or Fisher’s exact test, as appropriate.

^b^ *p*-Value for difference between HIV+ and HIV− urban participants using Chi-squared or Fisher’s exact test, as appropriate.

**Table 8.** Sociodemographic and household food security factors associated with HIV status of urban participants.

Variable		Odds ratio (95% CI^a^)	*p*-Value
Age		0.92 (0.90; 0.95)	<0.0001
Microwave oven in house	Yes vs. no	0.15 (0.06; 0.42)	0.0002
Vegetables easily available	Yes vs. no	0.43 (0.21; 0.89)	0.0224
Marital status	Married vs. not	0.20 (0.09; 0.41)	<0.0001
Money spent on food	Up to R50 vs. R101+	3.29 (1.58; 6.87)	0.0040
	R51–100 vs. R101+	1.22 (0.68; 2.20)	
Member of a church	Yes vs. no	0.22 (0.06; 0.76)	0.0167
Experienced death of spouse during past 12 months	Yes vs. no	4.91 (2.06; 11.73)	0.0003

^a^95% CI = 95% confidence interval.

#### Sociodemographic and household food security factors associated with HIV status in urban participants

In the urban sample, for every year that age increased, the odds of being HIV-infected decreased by 7%. HIV infection was negatively associated with being married (odds ratio 0.54), and positively associated with experiencing periods of food shortage (odds ratio 2.14). As in the rural sample, church membership was negatively associated with HIV-infection (odds ratio 0.46) (Table 9).

**Table 9.** Sociodemographic and household food security factors associated with HIV status of urban participants.

Variable		Odds ratio (95% CI^a^)	*p*-Value
Age		0.93 (0.91; 0.95)	<0.0001
Periods of food shortages	Yes vs. no	2.14 (1.19; 3.85)	0.0116
Marital status	Married vs. not	0.54 (0.33; 0.89)	0.0152
Member of a church	Yes vs. no	0.46 (0.23; 0.91)	0.0276

^a^95% CI = 95% confidence interval.

## Discussion

In both rural and urban areas, HIV-infected participants were significantly younger than their HIV-uninfected counterparts. According to the Centers for Disease Control and Prevention (CDC, 2012), a number of factors, such as social and economic factors including poverty, lack of access to healthcare, stigma and discrimination, contribute to the high levels of HIV in younger people. In the current study, the percentage of female participants was higher than male participants, possibly due to the fact that more women tend to stay home compared to men, and were therefore more easily available to participate. Women were also more likely to bring children to a research venue where free healthcare services were rendered.

With regard to sociodemographic indicators in this sample, HIV status was negatively associated with being married, probably due to an increased likelihood to have fewer sexual partners if married. The current study results showed that poverty was prevalent in both HIV-infected and HIV-uninfected groups, although HIV-infected persons in all areas were generally poorer and had a high level of food insecurity. This finding concurs with a study done by Bachmann and Booysen (2003) where households with an HIV-infected member were compared with unaffected neighbouring households in a rural and urban area in the Free State Province, South Africa. They also found that HIV-affected households tended to be poorer and have lower employment rates than unaffected households (Bachmann & Booysen, 2003).

HIV status among the rural participants in our study was negatively associated with having a microwave oven. Refrigerators and/or freezers, microwave ovens and television sets are more likely to be owned by higher-income individuals (Thompson & Sweaney, 1994). In this study, household income in rural areas differed significantly between HIV-infected and HIV-uninfected respondents. The same trend was seen in the urban group, although the difference in urban income was not significant. Sufficient income is necessary to ensure that enough food can be obtained to meet the requirements of a household and therefore income is an important determinant of a household's ability to meet food security needs (Coutsoudis et al., 2000; Hendriks, 2005).

An association between low income, food insecurity and nutrient inadequacies has been reported by Kirkpatrick and Tarasuk (2008). In the present study, expenditure on food in rural areas differed significantly between HIV-infected and HIV-uninfected participants with HIV-uninfected participants being more likely to spend more money on food. According to the International Food Security Assessment 2011–2021, food production at household level is important in assuring food security in sub-Saharan Africa (Barrett, 2010). Although not optimal, HIV-uninfected rural participants in this study were more likely to grow crops, have their own fruit trees and keep their own livestock compared to HIV-infected rural participants. In these rural participants, HIV infection was negatively associated with having access to vegetables from local farmers or shops, which is likely to result in improved household food security.

Household food insecurity was common in all communities included in this study, with the problem being worse in HIV-infected participants. In the urban sample of the current study, HIV status was positively associated with experiencing periods of food shortages, probably due to increased HIV infection in a situation of acute food insecurity (Gillespie & Kadiyala, 2005). In our study, periods of food shortage and hunger occurred commonly, especially among HIV-infected respondents. In the South African National Health and Nutrition Examination Survey of 2012, approximately 30% of school children indicated that they did not have food at home to put in their lunch boxes (2013).

Evidence from a study done by Kaschula (2011) in the north-eastern part of KwaZulu Natal, South Africa, suggests that households with both chronic poverty and chronic illness (with or without HIV/AIDS) were particularly prone to food insecurity. According to Hamelin, Habicht, and Beaudry (1999), coping strategies during times of food insecurity may include relying on credit to buy food, selling personal assets, skipping meals, limiting portion sizes, buying and preparing limited amounts of food, and parents depriving themselves to feed children (Hamelin et al., 1999). In the current study, coping strategies reported included asking family, neighbours or relatives for help (in terms of money or food) or borrowing money or food.

In this study, being a member of a church was negatively associated with HIV-infection in both samples. This could be ascribed to the support (both social and moral) offered in church groups. This support, which often includes food assistance, makes it less likely that these participants would turn to other ways of obtaining food or money such as transactional sex. A study in South Africa found a 35% rate of depression and a 15% rate of post-traumatic stress disorder among men and women with HIV-infection (Olley, Seedat, Nei, & Stein, 2004). Similarly, a study amongst persons with AIDS in South Africa found significantly higher rates of depression and anxiety in HIV-infected individuals (33%) than in uninfected participants (24%). In this study, more than half of all participants reported feeling sad, blue or depressed for two weeks or more in a row. Major stressors included loss of job, personal injury or illness, death or illness of another family member and food insecurity, especially amongst HIV-infected respondents.

We acknowledge there is a certain degree of bias regarding the age of rural volunteers in the study. Older and unemployed individuals were more likely to participate. More women than men participated in the study probably due to men being more likely to be employed as labourers, and therefore not available for interviews conducted during the day. It is also possible that ill persons might have been more likely to participate in the study where medical examinations were conducted due to limited health services, especially in rural areas. Due to these reasons, the authors acknowledge that the study group is probably not representative of the general population.

## Conclusions

HIV-infected persons in both rural and urban areas were generally poorer and had a high level of food insecurity. Indicators of lower socioeconomic status – such as not having a microwave oven, limited access to vegetables (except cabbage), a limited amount of money to buy food and experiencing periods of food shortage – were positively associated with HIV status. On the other hand, being married and being a member of a church was negatively associated with HIV status.

A vicious cycle emerges in that poverty promotes the spread of HIV/AIDS; and conversely, HIV/AIDS contribute to continuing poverty. Interventions that focus on poverty alleviation can make a substantial contribution to addressing HIV in South Africa. Measures to improve household food security in vulnerable communities are imperative. Finally, the social and moral support provided by church involvement has the potential to assist with both the physical (such as food aid) and emotional needs of vulnerable groups, including those that are HIV-infected.
